# ALS Cognitive Behavioral Screen-Phone Version (ALS-CBS™-PhV): norms, psychometrics, and diagnostics in an Italian population sample

**DOI:** 10.1007/s10072-021-05636-x

**Published:** 2021-10-02

**Authors:** Edoardo Nicolò Aiello, Antonella Esposito, Ilaria Giannone, Lorenzo Diana, Susan Woolley, Jennifer Murphy, Georgia Christodoulou, Lucio Tremolizzo, Nadia Bolognini, Ildebrando Appollonio

**Affiliations:** 1 PhD Program in Neuroscience, School of Medicine and Surgery, University of Milano-Bicocca, Monza, Italy; 2 Department of Psychology, University of Milano-Bicocca, Milan, Italy; 3 Sutter Pacific Medical Foundation, San Francisco, CA USA; 4 Biogen, Cambridge, MA USA; 5 Institute for Health Promotion and Disease Prevention Research, Keck School of Medicine, University of Southern California, Los Angeles, CA USA; 6 Neurology Section, School of Medicine and Surgery, University of Milano-Bicocca, Monza, Italy; 7 Neuropsychological Laboratory, IRCCS Istituto Auxologico Italiano, Milan, Italy

**Keywords:** Motor neuron disease, Frontotemporal degeneration, Telephone-based, Cognitive screening, Normative data, Psychometrics

## Abstract

**Background:**

Up to 50% of motor neuron disease (MND) patients show neuropsychological deficits which negatively affect prognosis and care. However, disability-related logistical issues and uneven geographical coverage of healthcare services may prevent MND patients from accessing neuropsychological evaluations. This study thus aimed to standardize for the Italian population the ALS Cognitive Behavioral Screen-Phone Version (ALS-CBS™-PhV), an MND-specific, telephone-based screening for frontotemporal dysfunction.

**Methods:**

The cognitive section of the ALS-CBS™-PhV, the Italian telephone-based Mini-Mental State Examination (Itel-MMSE), and the Telephone Interview for Cognitive Status (TICS) was administered to 359 healthy individuals (143 males, 216 females; age, 52.7 ± 15.8; education, 13.1 ± 4.4). Norms were derived through equivalent scores. Validity, factorial structure, reliability, diagnostic accuracy, and item difficulty and discrimination were examined. Statistical equivalence between the telephone-based and in-person versions was tested.

**Results:**

ALS-CBS™-PhV measures were predicted by age and education. The ALS-CBS™-PhV reflected a mono-component structure, converged with Itel-MMSE and TICS scores (*r*_s_ = .23–.51) and was equivalent to its in-person format (*t* = .37; *p* = .72). Good internal (Cronbach’s *α* = .61), test–retest (ICC = .69), and inter-rater (ICC = .96) reliability was detected. High accuracy was found when tested against both the Itel-MMSE and the TICS (AUC = .82–89). Backward digit span items were the most discriminative.

**Discussion:**

The ALS-CBS™-PhV is a statistically solid screening test for frontotemporal disorders featuring MND. Its standardization allows for (1) improvements in tele-healthcare for MND patients, (2) epidemiological applications, and (3) effective assessments in decentralized clinical trials. The ALS-CBS™-PhV can be also suitable for assessing bedridden and visually impaired patients with motor disorders.

## Introduction

Since motor neuron diseases (MNDs) and frontotemporal (FT) degenerations are pathophysiologically related [6], neuropsychological deficits within the FT *spectrum* occur in up to 50% of MND patients [34].

Early detection of FT involvement in MND is crucial due to its unfavorable impact on patients’ prognosis and management [24]. Neuropsychological screening in MND patients has thus entered the customary clinical practice [40], with an ad hoc nosographic system [34] and the development of MND-specific diagnostic tools [33].

Among the latter, the ALS Cognitive Behavioral Screen (ALS-CBS™) [39] represents a guideline-recommended [34], I-level test. The psychometric and diagnostic properties of the ALS-CBS™ have been thoroughly demonstrated, along with its usability [21]. The ALS-CBS™ indeed accounts for motor disabilities (i.e., dysarthria and upper limb deficits) and specifically targets FT functions (i.e., dysexecutive and frontal behavioral disorders) [40]. In particular, the ALS-CBS™ includes (1) a cognitive section, which assesses linguistically mediated/non-mediated executive functioning via motor-free tasks, as well as (2) a proxy report questionnaire covering the full range of FT behavioral changes. An Italian standardization has been recently provided [36].

Due to its brevity and minimal reliance on visual/physical supports, the English ALS-CBS™ has been successfully adapted to be administered over the telephone (ALS-CBS™-Phone Version, ALS-CBS™-PhV) [14]. In Italy, telemedicine has been shown to be promising in the clinical management of MND patients since it circumvents logistical issues related to motor disabilities and unequal geographical coverage of healthcare services [8, 38]. However, the full potential of diagnostic tele-neurology for MND patients has yet to be fully explored [5], especially with regard to neuropsychological evaluation.

Telephone-based neuropsychological assessment represents an evidence-based medium [9, 18] to reach populations that have difficulties accessing in-person visits [10].

Given the above premises, this study aimed at (1) adapting the ALS-CBS™-PhV to the Italian language, (2) testing the psychometric and diagnostic properties of the cognitive section, and (3) deriving normative data from a representative Italian population sample.

## Methods

### Participants

Three hundred fifty-nine healthy individuals were recruited from different regions of Italy (see Tables 1 and 2). Exclusion criteria were (1) history of neurological and/or major psychiatric diseases; (2) organ failures, non-compensated metabolic disorders, and severe internal conditions; and (3) uncorrected hearing deficits. Mild-to-moderate, corrected vision deficits were not addressed as an exclusion criterion.

**Table 1:** Sample stratification for age, education, and sex

	Age (M/F)							
Education	35 ≤	36–45	46–55	56–65	66–75	76–80	≥ 81	Total
5 ≤	0/0	0/0	0/1	0/0	2/6	1/8	3/4	6/19
6–8	1/1	0/6	5/13	10/11	4/7	1/4	3/1	24/43
9–13	17/7	2/6	21/33	17/24	6/4	2/3	0/0	65/77
14–18	6/8	2/1	0/6	1/2	0/0	0/0	0/0	9/17
≥ 19	11/18	3/4	12/17	11/15	1/3	1/2	0/1	39/60
Total	35/34	7/17	38/70	39/52	13/20	5/17	6/6	143/216

Cells show male/female (M/F) ratio for each co-occurrence

**Table 2:** Demographic and cognitive data

N		359
Age (years)		52.7 ± 15.75 (18–89)
Sex (M/F)		143/216
Education (years)		13.06 ± 4.44 (0–26)
N for Italian regions	North Italy	260
	Center Italy	17
	South Italy	82
N for occupation	White-collar	158
	Blue-collar	201
Itel-MMSE		21.51 ± .96 (14–22)
TICS		34.87 ± 3 (22–41)
ALS-CBS™-PhV	Total score	16.92 ± 2.7 (7–20)
	Attention	4.29 ± .98 (1–5)
	Concentration-WM	6.18 ± 1.65 (1–8)
	Concentration-Total	4.4 ± .81 (1–5)
	Tracking/Monitoring	4.21 ± 1.11 (0–5)
	Initiation and Retrieval	4.01 ± 1.06 (0–5)

*N*, number of participants; *M*, male; *F*, female; *Itel-MMSE*, Italian telephone-base Mini-Mental State Examination; *TICS*, Telephone Interview Cognitive Status; *ALS-CBS™-PhV*, ALS Cognitive Behavioral Screen-Phone Version; *WM*, working memory

The study was approved on behalf of the ethical committee of the University of Milano-Bicocca. Participants provided informed consent to participate in the study.

### Materials

The cognitive section of the ALS-CBS™ encompasses four subtests: (1) *Attention,* comprising oral command (*Commands*, 1a), syllable segmentation (*Mental Addition/Language*, 1b), and saccade/anti-saccade (*Eye Movements*, 1c) tasks; (2) *Concentration*, a backward digit span task; (3) *Tracking/Monitoring*, comprising backward month production (*Months*, 3a), forward alphabet production (*Alphabet*, 3b), and letter–number alternation (*Alternation Task*, 3c) tasks; and (4) *Initiation and Retrieval*, a phonemic verbal fluency task. Each subtest ranges 0–5 and the total ranges from 0–20.

In the telephone-based format, (1) *Commands* (1a) has been adapted to actions that can be performed by the patient and are audible by the examiner, and (2) the *Eye Movements* (1b) has been replaced with a motor-mediated task requiring the examinee to detect a verbal target among distractors. The original ranges have been maintained.

Items 1a and 1b were translated into Italian by a bilingual author and then back-translated to English by two other independent, bilingual, authors blinded to each other’s translations. No major discrepancies were detected. Remaining items were derived from the original, back-translated Italian ALS-CBS™ [36].

The behavioral section of the ALS-CBS™-PhV mirrors its de visu format but is delivered verbally over the telephone; it comprises fifteen 3-point Likert items (range, 0–45) questioning caregivers on patients’ behavioral changes (3 = “no change”; 0 = “large change”) and 4 “yes/no” questions on anxiety and depression.

The Italian telephone-based Mini-Mental State Examination (Itel-MMSE) [29] and the Telephone Interview for Cognitive Status (TICS) [19] were also administered as convergent measures of global cognition. Their total score range is 0–22 and 0–41, respectively. Validity and reliability evidence for both tests have been previously provided for the Italian population [16, 37].

The Italian ALS-CBS™-PhV will be provided upon request to the corresponding author (E. N. A.).

### Procedures

Before test administration, a detailed sound-check from both the examiner and the examinee standpoint was carried out to ensure good quality on the call. The examiner also made sure that the examinee was able to carry out those actions required to perform the tasks (e.g., pressing a key on the telephone pad) by, otherwise, instructing her/him. A third person was required to secure that the administration setting was free of facilitating elements (e.g., a calendar/watch providing the examinee with suggestions for temporal orientation items), as well as to confirm the correctness of address information needed to assess spatial orientation.

The ALS-CBS™-PhV was re-administered 30 days after the baseline to *N* = 126 participants (58 males, 68 females; age, 45.8 ± 14.66, 24–82; education, 14.9 ± 3.84, 0–26) to assess test–retest reliability. To test inter-rater reliability, two independent examiners scored *N* = 58 protocols (22 males, 36 females; age, 55.03 ± 11.03, 21–89; education, 13.14 ± 4.78, 5–25) blinded to each other’s ratings.

Twenty-six participants (12 males, 14 females; age, 42.2 ± 17.9, 19–80; education, 12.3 ± 3.28, 5–16) were administered both the in-person and the telephone-based ALS-CBS™ at a 14-day interval to determine their comparability. To control for carry-over effects, the administration order was counterbalanced across participants (*N* = 13 being administered first the paper-and-pencil ALS-CBS™ and then the telephone format, *N* = 13, and vice versa).

### Statistical analyses

Analyses were performed via SPSS 27 [25], R 4.0.1 (https://cran.r-project.org) and jamovi 1.6 [35].

A power analysis for multiple regressions was run through the R package *pwr* [12] accordingly to previous normative studies [36], yielding an a priori *N* of 347 as sufficient to achieve a 95% power (with *α* = .05, *f*^2^ = .05 and *df*_numerator_ = 3) [31].

Based on raw data distributing normally or not (the last scenario being indexed by skewness and kurtosis values ≥ |1| and |3|, respectively) [27], either parametric or non-parametric techniques were adopted to test associations of interest between continuous measures. Bonferroni corrections were applied when relevant.

Validity was assessed by convergence and at the structure level (principal component analysis). Reliability was assessed as internal consistency (Cronbach’s α), test–retest, and inter-rater — both via intra-class correlation coefficients.

Accuracy was tested via receiver operating characteristics analyses by addressing performances below vs. above the 5th percentile of the sample on the Itel-MMSE and the TICS as proxy reference measures.

Equivalence between the telephone-based and the paper-and-pencil in-person ALS-CBS™ was tested via a two one-sided test (TOST) procedure for paired-sample *t* tests [26], which allows determining whether the effect size of a between-mean difference is equivalent to zero.

By addressing global cognition as the latent trait, difficulty and discrimination were examined for each item by running a two-parameter, logistic item response theory model [3, 22] via the R package *mirt* [11].

Norms were derived through the equivalent score (ES) method [7] by adjusting raw scores for significant demographic confounders via regression-based equations, identifying outer/inner tolerance limits (oTL and iTL, respectively) and ES thresholds on ranked adjusted scores (ASs) and allotting them into a 5-level, quasi-continuous scale (ASs ≤ oTL → ES = 0, “defective”; oTL < ASs ≤ *Mdn* → ESs = 1, 2, and 3, “borderline,” “low-end normal,” and “normal,” respectively; ASs > *Mdn* → ES = 4, “high-end normal”). ES-related computation was carried out according to Aiello and Depaoli [1].

With respect to the *Concentration* subtest, normative values were computed for both the “working memory” (i.e., the span, ranging 0–5) and the “total” outcome (i.e., the number of correct sequences, ranging 0–8) — the former assessing working memory capacity, the latter being a measure of sustained attention during the execution of the task according to Pasotti et al. [30].

## Results

Cognitive scores are summarized in Table 2.

Acceptability rate was 100%. No clear floor/ceiling effects were detected.

ALS-CBS™-PhV total (*r*_*s*_(359) = −.46; *p* < .001) and subtest (−.43 ≤ *r*_*s*_(359) ≤ −.26; *p* ≤ .001) scores proved to be negatively related to age whereas positively to education (total, *r*_*s*_(359) = .52; *p* <.001; subtest, .34 ≤ *r*_*s*_(359) ≤ .41; *p* ≤ .001); no sex differences were detected (.34 ≤ *p* ≤ .87).

Both Itel-MMSE (*r*_*s*_(359) = .23; *p* < .001) and TICS (*r*_*s*_(359) = .51) scores were associated with the ALS-CBS™-PhV (*r*_*s*_(359) = .23; *p* < .001 and *r*_*s*_(359) = .51; *p* < .001, respectively); moreover, the ALS-CBS™-PhV subtests were all internally related (.23 ≤ *r*_*s*_(359) ≤ .87; *p* ≤ .001 at *α*_adjusted_ = .003).

The TOST procedure showed that the telephone-based and the in-person ALS-CBS™ were statistically equivalent (*t*(24) = .37; *p* = .718; in-person, 17.1 ± 2.5; telephone-based, 17.2 ± 3).

The ALS-CBS™-PhV showed acceptable internal consistency (Cronbach’s α for the four subtests = .61), moderate test–retest (ICC = .69), and excellent inter-rater (ICC = .96) reliability.

A mono-component structure underlying the four subtests was detected (46.65% of variance explained; .64 ≤ *r* ≤ .75), reflecting executive functioning efficiency. Item sensitivity and discriminative capability are reported in Table 3. *Concentration* and item overall showed mild-to-moderate difficulty; the most discriminative ones proved to be the last 6 items of backward digit span sequences (*Concentration*) and, to a lesser extent, the *Alternation Task*.

**Table 3:** Item difficulty and discrimination for the ALS-CBS™-PhV

Subtest	Item	Difficulty	Discrimination
Attention	Commands, 1	3.41^†^	1.12
	Commands, 2	2.07	.66
	Mental addition/language, 1	1.34	1.27
	Mental addition/language, 2	1.51	1.13
	Correct taps	2.66	.82
	^*●*^Incorrect taps	2.87	.3
Concentration	Sequence 1	-	-
	Sequence 2	4.25^†^	.83
	Sequence 3	3.48	1.83^**^
	Sequence 4	4.71^†^	2.43^**^
	Sequence 5	.98	1.58^*^
	Sequence 6	2.22	2.36^**^
	Sequence 7	− .61	2.37^**^
	Sequence 8	− .14	1.96^**^
Tracking Monitoring	^*●*^Months	3.22	.88
	Alphabet	3.23	1.22
	^*●*^Alternation task	1.27	1.46
Initiation and Retrieval	^*●*^Correct words	2.76	1.17
	^*●*^Errors	2^†^	.27

Higher values correspond to higher sensitivity and discriminative capability of items. The usual range of difficulty goes from − 4 to 4. [3, 22]. As for discrimination, items were classified as either “discriminative” (≥ 1.5) or “highly discriminative” (≥ 1.7) [3]. **†,** difficult; *, high discrimination; **, very high discrimination [3, 22]. ^●^Non-dichotomous items (dichotomized according to the 5th percentile of ranked raw scores). *Sequence 1* was dropped from the analysis as having 0 variance

When tested against a performance below vs. above the 5th percentile on the Itel-MMSE and the TICS, the ALS-CBS™-PhV showed high accuracy (Itel-MMSE, AUC = .82, 95% CI [.73, .91], *SE* = .05; TICS, AUC = .89, 95% CI [.83, .96], *SE* = .03).

Within multiple regression procedures, only transformed age and education proved to be simultaneous predictors of both ALS-CBS™-PhV total and subtest scores (age, |2.95|≤*t*≤|5.72|; *p* ≤ .005; education, |4.25|≤*t*≤|8.22|; *p* ≤ .001). Adjustment equations for ALS-CBS™-PhV total and subtest scores are reported in Table 4. A freely accessible, online applet for the automated computation of ASs based on age and education (https://enaiello.shinyapps.io/ALSCBSPhV/) was implemented via the R package *shiny* [13]. Furthermore, an offline score-sheet will be made available upon request to the corresponding author (E. N. A.).

**Table 4:** Adjustment equations for ALS-CBS™-PhV raw total and subtest scores

	Adjustment equations
Total score	AS = RS − 2.036709*[ln(100 − age) − 3.791907] − 2.700291*[ln(education) − 2.497723]
Attention	AS = RS + .000159*[(age^2) − 3024.637883] − .355773*[sqrt(education) − 3.55473]
Concentration-WM	AS = RS + .000001*[(age^3) − 185,105.752089] − .628171*[ln(education) − 2.497723]
Concentration-Total	AS = RS + .000002*[(age^3) − 185,105.752089] − 1.259983*[ln(education) − 2.497723]
Tracking/Monitoring	AS = RS + .000001*[(age^3) − 185,105.752089] − .86209*[ln(education) − 2.497723]
Initiation and Retrieval	AS = RS − .496252*[ln(100 − age) − 3.791907] − .414521*[sqrt(education) − 3.55473]

*M*, male; *F*, female; *AS*, adjusted score; *RS*, raw score; *ALS-CBS™-PhV*, ALS Cognitive Behavioral Screen-Phone Version; *WM*, working memory

Table 5 displays TLs and ES thresholds.

**Table 5:** Equivalent Scores for ALS-CBS™-PhV adjusted total and subtest scores

			Equivalent scores				
	oTL	iTL	0	1	2	3	4
ALS-CBS™-PhV-total	12.42	13.55	≤ 12.42	12.43–14.07	14.08–15.86	15.87–17.13	≥ 17.14
ALS-CBS™-PhV-attention	1.77	2.85	≤ 1.77	1.78–3.18	3.19–4.01	4.02–4.49	≥ 4.5
ALS-CBS™-PhV-concentration-WM	2.8	3.05	≤ 2.8	2.81–3.47	3.48–3.94	3.95–4.66	≥ 4.67
ALS-CBS™-PhV-concentration-total	2.8	3.98	≤ 2.8	2.81–4.31	4.32–5.42	5.43–6.34	≥ 6.35
ALS-CBS™-PhV-tracking/monitoring	1.88	2.54	≤ 1.88	1.89–2.77	2.78–3.73	3.74–4.59	≥ 4.6
ALS-CBS™-PhV-initiation and retrieval	1.84	2.69	≤ 1.84	1.85–2.81	2.82–3.58	3.59–4.09	≥ 4.1

*oTL*, outer tolerance limit; *iTL*, inner tolerance limit; *ALS-CBS™-PhV*, ALS Cognitive Behavioral Screen-Phone Version; *WM*, working memory

## Discussion

The present work provides Italian practitioners with a standardized telephone-based screening tool for FT cognitive disorders among MND patients. The cognitive section of the ALS-CBS™-PhV demonstrated (1) good convergent validity with established telephone-based measures of global cognition; (2) statistical equivalence with its paper-and-pencil format; (3) a clear mono-component structure (i.e., executive functioning-related cognitive efficiency); (4) moderate-to-excellent internal, test–retest, and inter-rater reliability; and (5) good accuracy in discriminating high vs. low levels of cognitive efficiency. Statistically sound norms for the ALS-CBS™-PhV and its subtests are also provided — along with an open-source applet for the automated computation of regression-adjusted scores. Finally, item-level information has been herein enclosed to help practitioners interpret test scores [2].

To the best of the authors’ knowledge, the present work is the first ever to fully standardize a telephone-based cognitive screening test in Italy, thus relevantly contributing to the growing evidence worldwide on the feasibility of tele-neuropsychological assessment [18].

The same cut-offs for the in-person ALS-CBS™ version, provided by Tremolizzo et al. [36], should be used for the behavioral section of the ALS-CBS™-PhV, adapted to Italian; however, further investigations should be carried out for ensuring its feasibility over the telephone.

The availability of a brief, normed, statistically robust, MND-specific telephone-based screening for neuropsychological deficits allows for clear improvement in testing within both healthcare and research settings. From a clinical standpoint, the ALS-CBS™-PhV will help deliver I-level neuropsychological assessment to difficult-to-reach patients, as well as to monitor their cognitive status by overcoming logistical issues. Such an option is particularly important when for providing healthcare to vulnerable populations in the COVID-19 era; MND patients, indeed, often suffer from disease-related respiratory impairments. Additionally, underserved populations can benefit from large-scale, telephone-based neuropsychological screening [17], which can represent the early stage of a multi-phasic, population-based diagnostic process [15].

From an experimental standpoint, the ALS-CBS™-PhV may allow large-scale epidemiological studies on FT disorders in MND patients [23] and facilitate follow-ups in clinical trials [4], specifically when combined with the available, self-administered ALS Functional Rating Scale - Revised [28].

By assessing executive functioning, the ALS-CBS™-PhV can furthermore be feasible for testing patients with frontotemporal dementias or other motor diseases possibly presenting with a dysexecutive profile (e.g., *extra*-pyramidal disorders) — as shown for another MND-specific I-level neuropsychological tool, the Edinburgh Cognitive and Behavioral ALS Screen [20, 32].

Moreover, by requiring minimal physical supports and not relying on visual elements, the ALS-CBS™-PhV can be adopted for bedside evaluations and the in-person assessment of patients with visual impairments.

Finally, it has to be noted that future studies are needed to test the clinical usability of the ALS-CBS™-PhV in both cross-sectional and longitudinal dimensions. With this respect, it should be borne in mind that the ALS-CBS™-PhV appears to be applicable only to patients with sufficiently spared hand movements and intelligible speech.

Future developments might also focus on the feasibility of a videoconference-based format of the ALS-CBS™, which could represent a valid alternative for patients who cannot/are unwilling to undergo a telephone-based assessment [10].

## Data Availability

Part of the data collected and analyzed within the present study are openly accessible at https://osf.io/k7f98/. Data regarding the Telephone Interview for Cognitive Status could not be made publicly accessible as being under the copyright of Giunti Psychometrics.
