# Correction to: ALS Cognitive Behavioral Screen‑Phone Version (ALS‑CBS™‑PhV): norms, psychometrics, and diagnostics in an Italian population sample

**DOI:** 10.1007/s10072-022-06293-4

**Published:** 2022-07-23

**Authors:** Edoardo Nicolò Aiello, Antonella Esposito, Ilaria Giannone, Lorenzo Diana, Susan Woolley, Jennifer Murphy, Georgia Christodoulou, Lucio Tremolizzo, Nadia Bolognini, Ildebrando Appollonio

**Affiliations:** 1 PhD Program in Neuroscience, School of Medicine and Surgery, University of Milano-Bicocca, Monza, Italy; 2 Department of Psychology, University of Milano-Bicocca, Milan, Italy; 3 Sutter Pacific Medical Foundation, San Francisco, CA USA; 4 Biogen, Cambridge, MA USA; 5 Institute for Health Promotion and Disease Prevention Research, Keck School of Medicine, University of Southern California, Los Angeles, CA USA; 6 Neurology Section, School of Medicine and Surgery, University of Milano-Bicocca, Monza, Italy; 7 Neuropsychological Laboratory, IRCCS Istituto Auxologico Italiano, Milan, Italy

**Correction to: Neurological Sciences (2022) 43:2571–2578**

**https://doi.org/10.1007/s10072-021-05636-x**

Missing funding information has been added in the Funding Note.

**Funding Note** Research partially funded by the Italian Ministry of Health to N.B.

